# Clinical and Histopathological Features of Oral Verruciform Xanthoma: A Case Series of Three Patients

**DOI:** 10.1002/ccr3.72526

**Published:** 2026-04-20

**Authors:** Taku Kimura, Ken‐ichiro Sakata, Keisuke Nakamura, Aya Yanagawa Matsuda, Kazuhiro Kawamura, Yusuke Nakamura

**Affiliations:** ^1^ Department of Oral Diagnosis and Medicine Hokkaido University Graduate School of Dental Medicine Sapporo Hokkaido Japan; ^2^ Department of Oral and Maxillofacial Surgery Sunagawa City Medical Center Sunagawa Hokkaido Japan; ^3^ Vascular Biology and Molecular Pathology, Hokkaido University Graduate School of Dental Medicine Sapporo Hokkaido Japan; ^4^ Department of Diagnostic Pathology Faculty of Medicine, Oita University Yufu Oita Japan

**Keywords:** carcinoma, chronic, gingiva, graft versus host disease, macrophage, oral mucosal lesions, verruciform xanthoma, verrucous

## Abstract

Oral verruciform xanthoma (OVX) is a rare benign lesion of the oral mucosa that often mimics other oral diseases. We report three cases of OVX and describe their clinical and histopathological characteristics. All patients presented with asymptomatic, well‐demarcated verrucous lesions located on the attached gingiva. The patients ranged in age from 59 to 85 years, with a median age of 73 years. One patient had a history of umbilical cord blood transplantation and possible chronic graft‐versus‐host disease. Clinically, the lesions were suspected to represent papillary lesions. Excisional biopsy was performed in all cases. Histopathological examination demonstrated papillary epithelial architecture with elongated rete ridges and infiltration of foamy macrophages within the underlying connective tissue, thereby confirming the diagnosis of OVX. No recurrence was observed during the follow‐up period. These findings emphasize the importance of histopathological evaluation for the accurate diagnosis of OVX.

## Key Clinical Message

Oral verruciform xanthoma is a rare benign oral lesion, sometimes clinically misdiagnosed as verrucous carcinoma or squamous cell carcinoma because of its verrucous or papillary appearance. Owing to its variable clinical presentation, accurate diagnosis requires histopathological identification of foamy macrophages. Surgical excision is an effective treatment, with a favorable prognosis and low risk of recurrence.

## 1 Introduction

Oral verruciform xanthoma (OVX) is a rare, benign, solitary lesion arising from the oral mucosa, with a reported incidence of 0.025%–0.095%, and it was first described by Shafer in 1971 [1, 2]. OVX most commonly occurs in the masticatory mucosa, particularly the attached gingiva and hard palate, which together account for approximately 70% of reported cases, followed by the tongue, floor of the mouth, and lip mucosa [3]. Although the pathogenesis of OVX remains unclear. Several factors have been suggested to contribute to its development, including chronic inflammations, lipid metabolism disorders, chronic graft‐versus‐host disease (cGVHD), and genetic factors [4, 5, 6, 7, 8, 9]. OVX is generally asymptomatic and exhibits indolent growth, and typically presents as well‐demarcated white, red and/or yellow‐colored patches with a papillary or verrucous surface [10]. Its clinical presentation may resemble leukoplakia, one of the oral potentially malignant disorders, as well as malignant tumors such as squamous cell carcinoma (SCC) and verrucous carcinoma (VC) [11]. Therefore, OVX is sometimes clinically misdiagnosed as other papillary or verrucous oral lesions, particularly VC [12]. Histologically, OVX is characterized by papillary or verruciform epithelial architecture and the presence of foamy macrophages, xanthoma cells, within the connective tissue, typically without cytologic atypia [12]. Therefore, it is essential to distinguish OVX from these lesions through histopathological evaluation. This case series reports three patients with OVX and aims to review the etiological features associated with OVX.

## 2 Case History/Examination

**FIGURE 1:**
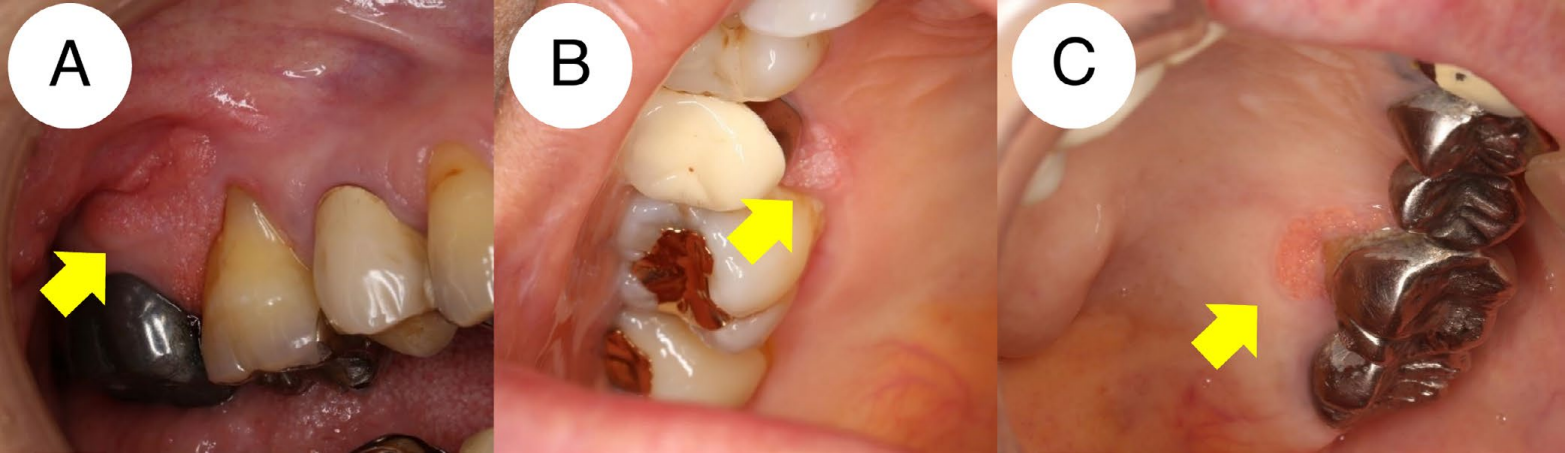
Clinical manifestations of three patients. The patients displayed verrucous‐surfaced and well‐demarcated patches (A, B, and C) on the attached gingiva. Each solitary lesion was asymptomatic, soft, and non‐tender on palpation. Arrows indicate the lesion area.

Three cases were included in this report (Table 1). The patients comprised two females and one male, aged 59–85 years (median, 73 years of age). All patients were of Japanese ethnicity. One patient reported a history of former smoking and regular alcohol consumption. Medication history included the use of an anti‐hypertensive drug in one patient. At the first visit to the Department of Oral Diagnosis and Medicine at Hokkaido University Hospital, a well‐defined lesion with a rough surface was detected in the attached gingiva in all three patients (Figure 1A−C). Among them, all the patients showed sessile lesions. In all cases, the lesions were located adjacent to dental prostheses. One patient had undergone umbilical cord blood transplantation for acute myeloid leukemia 7 years prior to her first visit to our department.

**TABLE 1:** Summary of our three patients with oral verruciform xanthoma.

Patient No.	Age at the first visit (years old)	Sex	Background				Clinical features					Follow‐up period (postoperative; months)
			Smoking	Alcohol	Medical history	Medication	Location #universal numbering system	Duration of the lesion prior to the first visit (months)	Color	Size (mm)	Surface appearance	
1	59	F	Never	Never	Acute myeloid leukemia; Ulcerative colitis	Brotizolam	Gingiva, #3	3	Red	10	Verrucous	84
2	73	M	Former	Regular	“Hypertension; Angina pectoris; Cerebrovascular disorder; Hyperuricemia; Glaucoma; Colon polyps”	Lansoprazole; Febuxostat; Cilostazol; Amlodipine	Gingiva, #4	1	White	2	Verrucous	1
3	85	F	Never	Never	Cerebral infarction	Clopidgrel; Esomeprazole; Mosapride; Alprazolam	Gingiva, #14	1	Pink	10	Verrucous	1

## 3 Methods

All the patients were asymptomatic, and panoramic X‐ray examination did not reveal any apparent bone changes adjacent to the lesions. The initial clinical diagnosis was a papillary lesion suspicious for squamous papilloma accompanied by inflammation. The differential diagnosis included VC and OVX. All patients underwent surgical resection with a 1 mm safety margin, and the resection was performed above the periosteum.

## 4 Conclusion and Results

**FIGURE 2:**
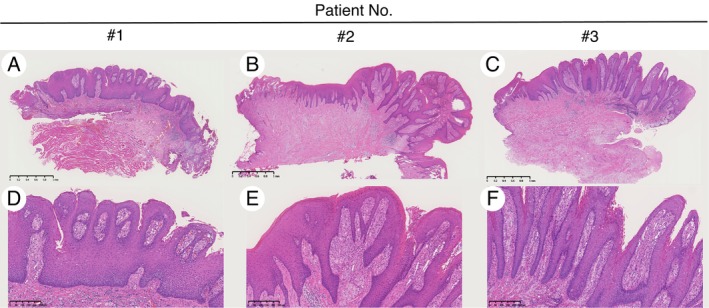
Histopathological findings of OVX in three patients. Each patient exhibited hyperplastic papillary architecture in the squamous epithelium and elongated rete ridges extending toward the connective tissue (A–C, hematoxylin and eosin staining, original magnification). Foamy macrophages (xanthoma cells) were confined to the connective tissue papillae without deeper extension into the lamina propria. Prominent surface parakeratosis with focal parakeratin clefting between papillary projections, neutrophilic exocytosis within the parakeratin layer, and mild‐to‐moderate chronic inflammatory cell infiltration in the underlying connective tissue were also observed (D–F, hematoxylin and eosin staining, original magnification ×400).

Histopathological examination revealed hyperplastic papillary architecture in the squamous epithelium, elongated rete ridges extending toward the connective tissue, and infiltration of foamy macrophages, leading to diagnosis of OVX in all patients (Figure 2A−F). No evident dysplastic changes were detected in the specimens. The median follow‐up period was 1 month (range, 1–84 months) after the surgery, and all the patients showed no recurrence during follow‐up.

## 5 Discussion

### 5.1 Epidemiology and Clinical Background

This case series reviewed three patients with OVX, all of whom were successfully treated surgically without recurrence. Our patients had no notable medical history previously reported to be associated with OVX development, except for one patient who may have developed cGVHD following umbilical cord blood transplantation, which carries a lower risk of GVHD than hematopoietic stem cell transplantation [13, 14]. To date, some OVXs have been reported in association with systemic diseases including endocrine or lipid metabolism disorders such as diabetes mellitus and hyperlipidemia [15, 16]. Additionally, chronic oral inflammatory diseases—including oral lichen planus, pemphigus vulgaris, and cGVHD—have been suggested to contribute to OVX development [15, 16]. Clinically, those OVXs associated with systemic disease often present in a multifocal form, while OVX generally occurs as a solitary lesion [9, 17]. Therefore, the lesion pattern may provide a clue for predicting whether an OVX is associated with an underlying disease. In particular, OVX associated with cGVHD has been reported to be higher prevalence (2.2%) compared with healthy individuals (0.04%) [7, 9]. To date, 13 cases of OVX related to the cGVHD following hematopoietic stem cell transplantation have been reported [8, 9]. OVX associated with cGVHD is typically asymptomatic, and approximately one‐third of the patients present with multiple OVX lesions (4/13 patients, 30.8%). In these 13 patients, lesions were located in the masticatory mucosa (6/13, 46.2%), buccal mucosa (4/13, 30.8%), and tongue (1/13, 7.7%), respectively [8, 9]. Our patient, who had received the cord blood transplantation 7 years prior to the first visit, developed OVX on the gingiva and did not exhibit any apparent oral symptoms related to cGVHD. Therefore, OVX should be considered among the potential oral manifestations when managing patients with cGVHD.

**FIGURE 3:**
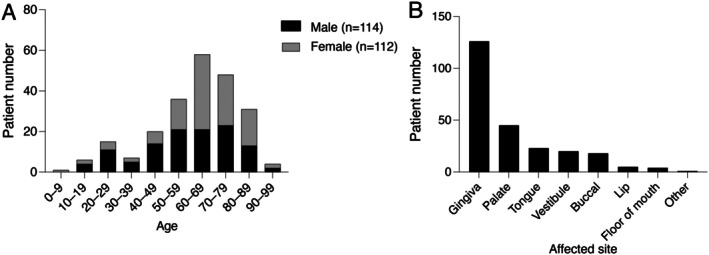
Etiology of oral verruciform xanthoma. No significant sex differences in OVX prevalence were observed, with the highest prevalence among the patients in their 60s (A). The gingiva (51.7%, 125/242) was the most frequently affected site, followed by the palate (18.6%, 45/242) and tongue (9.5%, 23/242), respectively (B).

To further understand the clinical profile of OVX, we combined our data with previous review data to analyze age distribution and lesion location. No significant sex differences were observed, and the highest prevalence was seen in patients in their 60s (Figure 3A) [12, 18]. Gingiva (51.7%, 125/242) was the most frequently affected site, followed by the palate (18.6%, 45/242) and tongue (9.5%, 23/242) (Figure 3B) [12, 18]. Overall, masticatory mucosa, including the gingiva and palate, accounted for 70.3% (170/242) of all cases.

### 5.2 Pathogenesis

The exact pathogenesis of OVX remains unclear and largely considered as reactive or related to local inflammation; however, previous studies have demonstrated that abnormal immune responses mediated by macrophages contribute to OVX development [6, 19]. Histologically, OVX is characterized by the infiltration of foamy macrophages, termed xanthoma cells, within the connective tissue [12]. The formation of xanthoma cells is thought to result from macrophages scavenging lipid substances released from the overlying epithelium in response to chronic inflammation [18]. At the molecular level, monocyte chemotactic protein‐1 (MCP‐1), one of the major chemokines, and its receptor C‐C chemokine receptor type 2 (CCR2) are key mediators of OVX development. Local inflammation initially stimulates squamous epithelial cells to increase lipid biosynthesis [20]. In addition, basal epithelial cells upregulate MCP‐1 secretion, which attracts CCR2‐positive macrophages [21, 22]. Activated T cells induced by inflammation modulate the production of both MCP‐1 and CCR2, thereby promoting further recruitment of CCR2‐positive macrophages into the connective tissue. Furthermore, MCP‐1 has been reported to induce scavenger receptors, such as macrophage scavenger receptor 1 (MSR1), on macrophages [23]. MSR1 allows macrophages to internalize low‐density lipoproteins (LDLs) produced by epithelial cells in response to chronic inflammation. Eventually, the macrophages engulf and oxidize LDLs, becoming lipid‐filled foamy macrophages, or xanthoma cells. These cells produce oxidized LDLs (ox‐LDLs), which act as chemoattractants for circulating monocytes and T cells. As a result, the persistent recruitment of MSR1‐expressing macrophages and continued production of ox‐LDLs contribute to the maintenance of OVX [3, 18, 24]. This hypothesis is also supported by immunohistochemical staining for CD68, a lysosomal glycoprotein specifically expressed in cells of the monocyte–macrophage lineage, and CD163, a macrophage‐specific scavenger receptor [25]. In our patients, all patients developed OVX adjacent to a dental crown. Although a relationship between dental prostheses and OVX development has not been established, dental prostheses may contribute to chronic mechanical irritation, provoking local inflammation in the oral mucosa.

### 5.3 Differential Diagnosis

Clinically, OVX exhibits a variety of surface features (papillary, verrucous, or flat) and color variation (ranging from white and yellow to red or pink), which often lead to misdiagnosis as other oral lesions such as papilloma, fibroma, verrucous hyperplasia, and even oral SCC and VC [7, 26]. Distinguishing OVX from malignant tumors is crucial to avoid unnecessary excessive surgical intervention. In particular, VC shares several clinical features with OVX, including indolent growth and papillary or verrucous appearance, and an asymptomatic course [27]. Therefore, histopathological examination is essential to differentiate OVX from other lesions.

Histologically, OVX is characterized by papillary or verrucous epithelial architecture without cytological atypia, accompanied by acanthosis, thickening of the prickle cell layer, and multiple elongated rete ridges [27]. However, these histopathological features are also observed in VC. The distinctive feature of OVX compared with VC includes (1) the presence of xanthoma cell infiltration in the underlying connective tissue without deeper extension into the lamina propria, and (2) the absence of bulbous epithelial downgrowth into the underlying connective tissue [27, 28]. Additional diagnostic hallmarks frequently observed in OVX include prominent surface parakeratosis with an orange hue, parakeratin clefting between papillary projections, and neutrophilic exocytosis within the parakeratin layer [18]. Notably, superficial surgical resection may fail to capture these diagnostic features; therefore, adequate surgical depth is recommended. In our patients, all lesions exhibited xanthoma cell infiltration in the underlying connective tissue (Figure 2A−F); thus additional immunohistochemical analysis was not performed.

### 5.4 Limitations

This case series: highlights the clinical and histopathological characteristics of OVX; however, several limitations should be acknowledged. Our three patients were followed at our department for a median of 1 month (range, 1–84 months) after surgery. One patient was monitored regularly up to 84 months due to OVX suspected to be associated with cGVHD. During her long‐term follow‐up period, she did not experience recurrence of OVX or other cGVHD‐related mucosal lesions. In contrast, the remaining two patients were followed for less than 1 year. This follow‐up period in our report is relatively short compared with reported recurrence intervals. Literature from large series indicates that recurrence is rare (0%–12% in some reports including 6/429 [1.4%] in Tamiolakis and 3/212 [1.4%] in Belknap, often reported without full follow‐up data) but typically occurs 2–5 years post‐excision (mean: ~2.8 years in some reports), with meaningful assessment requiring longer follow‐up (mean follow‐up often 3–9 years) [7, 12]. No recurrence was observed during the limited follow‐up period in our series; however, longer surveillance is recommended given the reported recurrence intervals.

## 6 Conclusion

OVX is a rare oral lesion that often shares clinical features with oral malignancies, including SCC and VC. Accurate diagnosis requires histopathological evaluation, with hallmarks including foamy macrophages (xanthoma cells) in the underlying connective tissue, absence of cytological atypia, and lack of bulbous epithelial downgrowth. Adequate‐depth biopsy is essential to capture these diagnostic features and avoid misdiagnosis. This case series emphasizes that OVX may be associated with chronic inflammation, including cGVHD, underscoring the importance of long‐term follow‐up, as recurrence can occur several years after surgical resection. Clinicians should consider OVX in the differential diagnosis of papillary or verrucous oral lesions, particularly in older patients or those with systemic or inflammatory comorbidities. Further studies are needed to elucidate the precise pathogenic mechanisms, evaluate recurrence risk factors, and establish standardized management protocols. By integrating clinical, histological, and etiological insights, this report aims to facilitate accurate recognition and appropriate management of OVX in routine clinical practice.

## Author Contributions

**Taku Kimura:** conceptualization, data curation, formal analysis, investigation, methodology, resources, software, visualization, writing – original draft. **Ken‐ichiro Sakata:** conceptualization, funding acquisition, project administration, resources, supervision, validation, visualization, writing – review and editing. **Keisuke Nakamura:** data curation, investigation, resources, validation, visualization, writing – review and editing. **Aya Yanagawa Matsuda:** investigation, methodology, resources, validation, visualization, writing – review and editing. **Kazuhiro Kawamura:** conceptualization, methodology, project administration, supervision, validation, visualization, writing – review and editing. **Yusuke Nakamura:** data curation, methodology, resources, visualization, writing – review and editing.

## Funding

The authors have nothing to report.

## Disclosure

**Human subjects**: Consent was obtained by all participants in this study. **Payment/services info:** All authors have declared that no financial support was received from any organization for the submitted work. **Financial relationships:** All authors have declared that they have no financial relationships at present or within the previous 3 years with any organizations that might have an interest in the submitted work. **Other relationships:** All authors have declared that there are no other relationships or activities that could appear to have influenced the submitted work.

## Consent

Written informed consent was obtained from all patients for publication of this case series and accompanying images.

## Conflicts of Interest

The authors declare no conflicts of interest.

## Data Availability

The data are not publicly available due toxxx3000; patient privacy concerns but are available from the corresponding author upon reasonable request.
